# Beyond the Spotlight: *Enterobacter* spp. as Overlooked Carbapenemase Producers in Europe

**DOI:** 10.3390/antibiotics14101045

**Published:** 2025-10-18

**Authors:** Ivana Cirkovic, Snezana Brkic

**Affiliations:** 1 Institute of Microbiology and Immunology, Faculty of Medicine, University of Belgrade, 11000 Belgrade, Serbia; 2 Department of Medical Microbiology, University Clinical Center of Serbia, 11000 Belgrade, Serbia; brkic.snezana@gmail.com

**Keywords:** *Enterobacter* spp., carbapenemases, Europe, AMR surveillance, ESKAPEE

## Abstract

Antimicrobial resistance (AMR) poses a critical global health challenge, with carbapenemase-producing *Enterobacterales* (CPE) representing one of the most urgent threats. While *Klebsiella pneumoniae* and *Escherichia coli* have been the focus of most surveillance programs, *Enterobacter* spp., members of the *Enterococcus faecium*, *Staphylococcus aureus*, *Klebsiella pneumoniae*, *Acinetobacter baumannii*, *Pseudomonas aeruginosa*, *Enterobacter* spp., and *Escherichia coli* (ESKAPEE) group, remain an underrecognized but increasingly important reservoir of carbapenemase genes in Europe. Despite being categorized by the World Health Organization (WHO) as “critical-priority” pathogens, *Enterobacter* spp. are largely excluded from major AMR surveillance frameworks, creating blind spots in detection and control. This review summarizes the taxonomy, intrinsic resistance mechanisms, and clinical relevance of *Enterobacter* spp., with a particular focus on carbapenemase epidemiology across Europe. We highlight the distribution and genetic context of major carbapenemases, including VIM, OXA-48-like, KPC, and NDM, and discuss emerging or minor enzymes such as IMI, FRI, GES, and IMP. Epidemiological data reveal shifting dominance patterns over time, with VIM enzymes consolidating their prevalence after 2015, while OXA-48-like and KPC declined, and NDM gained ground. The genetic diversity of *Enterobacter* spp., coupled with their ability to act as both nosocomial pathogens and silent intestinal or environmental reservoirs, facilitates the dissemination of carbapenemase genes via epidemic plasmids and clonal expansion. Addressing the growing impact of carbapenemase-producing *Enterobacter* spp. requires their systematic inclusion in national and international monitoring programs, expanded use of genomic epidemiology in clinical microbiology, and better alignment between research, clinical practice, and policy. A One Health approach is essential to curb the spread of carbapenemases across human, environmental, and animal reservoirs, and to safeguard the remaining therapeutic options.

## 1. Introduction

The rise of antimicrobial resistance (AMR) represents an unprecedented global health threat, with projections estimating that AMR could lead to 39 million deaths between 2025 and 2050–equivalent to one death every 20 s [1]. At the heart of this crisis lie the ESKAPEE pathogens: *Enterococcus faecium*, *Staphylococcus aureus*, *Klebsiella pneumoniae*, *Acinetobacter baumannii*, *Pseudomonas aeruginosa*, *Enterobacter* spp., and *Escherichia coli*. These bacteria are notorious for their ability to evade antibiotic action and are among the primary culprits of healthcare-associated infections [2,3]. Beyond being leading causes of nosocomial infections, they represent paradigms of resistance, pathogenesis, and transmission. Their mastery in “escaping” the lethal effects of antimicrobials underscores their prominent designation on the 2024 World Health Organization (WHO) Global Priority Pathogens List (GPPL) [3].

All seven ESKAPEE pathogens are ranked within the “critical” or “high-priority” tiers of the 2024 WHO GPPL, reflecting their extensive resistance to both frontline and last-resort antibiotics [3]. Among them, *A. baumannii*, *K. pneumoniae*, *Enterobacter* spp., *E. coli*, and *P. aeruginosa* stand out among Gram-negative bacteria due to their advanced resistance mechanisms, including the production of β-lactamases and the deployment of efflux pumps [4,5]. These molecular strategies have rendered formerly reliable treatments increasingly ineffective, drastically narrowing the range of therapeutic options available to clinicians [6].

Additionally, the ESKAPEE pathogens are responsible for a wide range of severe and often life-threatening infections, such as bloodstream infections, urinary tract infections, pneumonia, surgical site infections, and soft tissue infections [7]. Their persistence in healthcare settings worldwide, coupled with their exceptional adaptability, imposes a tremendous burden on healthcare systems. Although novel antibiotics and β-lactamase inhibitors have been introduced, the continuing threat posed by these organisms underscores the urgent need for bold innovation in both prevention and treatment strategies [8].

Recent evidence highlights the need for an integrated global response, prioritizing enhanced surveillance infrastructure, reductions in inappropriate antibiotic use, expanded antimicrobial stewardship programs, and strong international collaboration–to mitigate the AMR crisis and protect future generations from the onset of a post-antibiotic era [9].

AMR surveillance is a foundational component of global public health strategies, providing the critical data needed to monitor resistance patterns, shape informed policies, guide clinical practice, and tailor stewardship interventions [10]. It entails the continuous collection, analysis, and interpretation of resistance data from a broad range of bacterial species and infection settings.

Importantly, the WHO designates third-generation cephalosporin-resistant and carbapenem-resistant *Enterobacterales* as pathogens of critical priority, underscoring their severe threat to public health due to limited treatment options. Nevertheless, major AMR surveillance initiatives, including the Global Antimicrobial Resistance and Use Surveillance System (GLASS), the European Antimicrobial Resistance Surveillance Network (EARS-Net), and the Central Asian and European Surveillance of Antimicrobial Resistance (CAESAR) currently monitor resistance trends in only two *Enterobacterales* members of the ESKAPEE group: *K. pneumoniae* and *E. coli*. In contrast, *Enterobacter* spp., despite being part of the same critical-priority category and exhibiting high levels of AMR, remain largely excluded from routine AMR surveillance. This oversight allows *Enterobacter* spp. to slip under the global monitoring radar, posing an unrecognized but growing threat within healthcare settings.

## 2. Taxonomy and Clinical Relevance of *Enterobacter* spp.

*Enterobacter* spp. is a genus of Gram-negative non-spore-forming bacteria belonging to the *Enterobacterales* order, widely recognized as a cause of human infections, particularly in hospital settings [11]. In clinical microbiology laboratories, species-level identification is often carried out using phenotype-based methods, including automated systems such as Matrix-Assisted Laser Desorption Ionization–Time Of Flight Mass Spectrometry (MALDI-TOF MS). These systems typically identify isolates as *E. cloacae*, though occasionally as *E. asburiae*, *E. hormaechei*, or *E. kobei*. However, it is well established that phenotypic methods frequently misidentify *Enterobacter* spp. and lack reliability for accurate taxonomic resolution [12].

Although 16S rRNA gene sequencing has long been regarded as the cornerstone for bacterial taxonomy and phylogenetic classification, its discriminative power is limited within the *Enterobacter cloacae* complex (ECC) and related taxa. The high degree of sequence conservation among members of *Enterobacter* spp., often exceeding 98.5–99.0% similarity, leads to substantial overlap between species and even across genera such as *Klebsiella*, *Citrobacter*, and *Cronobacter* [13]. As a result, the 16S rRNA gene cannot reliably differentiate closely related ECC members such as *E. cloacae*, *E. hormaechei*, and *E. kobei* [14]. In addition, *Enterobacter* spp. genomes frequently contain multiple heterogeneous copies of the 16S rRNA gene, which may differ slightly in sequence composition. This intragenomic heterogeneity further reduces the resolution and can mislead species-level assignments when only one or a few copies are sequenced [15]. Such limitations are particularly critical in clinical microbiology, where accurate species identification has direct implications for AMR profiling and epidemiological tracing.

Given the high degree of genetic similarity among closely related taxa, modern taxonomic frameworks increasingly rely on higher-resolution molecular tools such as multilocus sequence typing (MLST) and whole-genome sequencing (WGS). These methods provide superior discriminatory power through the comparison of conserved housekeeping genes (e.g., *gyrB, rpoB, infB, atpD*) or whole-genome average nucleotide identity metrics [16,17]. WGS-based phylogenomics not only refines *Enterobacter* spp. taxonomy but also enables accurate assignment of isolates to specific clades or sequence types within the ECC, thereby overcoming the inherent limitations of 16S rRNA-based identification. As the cost of these sequencing technologies has significantly decreased, MLST and WGS has become increasingly integrated into clinical microbiology workflows, providing high-resolution and reliable species identification [18]. Accurate taxonomic classification, however, depends on continuously updated and curated genomic data. The taxonomy of *Enterobacter* spp. is inherently complex and has been further complicated by recent reassignments of several species to other genera (e.g., *E. aerogenes* to *Klebsiella aerogenes*, *E. agglomerans* to *Pantoea agglomerans*, and *E. cowanii* to *Kosakonia cowanii*). In 2020, Wu et al. conducted a comprehensive WGS-based taxonomic analysis that refined the classification of the genus, now comprising 22 validly recognized species [17]. Consequently, many strains previously identified as *E. cloacae* are now correctly recognized as *E. hormaechei* or related taxa based on genomic data [19].

The most clinically relevant species belong to the ECC, a group comprising multiple phylogenetically related species and subspecies, including but not limited to *E. cloacae*, *E. hormaechei*, *E. asburiae*, *E. ludwigii*, and *E. kobei*. Recent molecular-based analyses have delineated the ECC into as many as 18 distinct phylogenetic clusters (designated A-R), aligning with Hoffmann clusters I–XIII and several additional genomic groups. To date, over 1000 sequence types, specifically, 1069, have been identified, underscoring the remarkable genetic diversity within the complex [20]. *E. hormaechei* and *E. cloacae* predominate in clinical settings, representing about 40–48% and 25–42% of ECC isolates, respectively [21].

From a clinical perspective, ECC species are recognized as opportunistic pathogens and are commonly implicated in nosocomial infections. These include bloodstream infections, particularly in critically ill or immunocompromised individuals, as well as catheter-associated urinary tract infections, ventilator-associated pneumonia, surgical site infections, and device-related infections.

A major therapeutic challenge in managing infections caused by members of the ECC lies in their robust intrinsic resistance mechanisms. These organisms naturally produce chromosomally encoded AmpC β-lactamases, which confer baseline resistance to a broad range of β-lactam antibiotics, including ampicillin, amoxicillin–clavulanate, and first- and second-generation cephalosporins. Importantly, exposure to β-lactam agents can lead to derepression or hyperproduction of these enzymes, significantly increasing resistance levels and potentially resulting in clinical treatment failure [22]. In addition to enzymatic degradation, *Enterobacter* spp. exhibit reduced outer membrane permeability, a structural feature that impedes antibiotic entry. This is primarily due to the downregulation or loss of major porins such as OmpC and OmpF, which are essential for the passive diffusion of hydrophilic antibiotics. Loss of these porins can synergize with β-lactamase activity to confer high-level resistance even to carbapenems [23]. Further complicating treatment is the presence of chromosomally encoded multidrug efflux pumps, particularly the AcrAB-TolC system. This efflux system actively exports a wide array of antimicrobial compounds, including fluoroquinolones, tetracyclines, chloramphenicol, and some β-lactams [24]. Efflux-mediated resistance may be basal or inducible, and it is often enhanced in the context of envelope stress or global regulatory mutations affecting *marA* or *soxS* pathways. Moreover, *Enterobacter* spp. are intrinsically resistant to certain antimicrobial classes due to the impermeability of their outer membrane and lack of suitable targets. Glycopeptides like vancomycin, for instance, are ineffective due to their inability to penetrate the Gram-negative cell envelope. Similarly, macrolides and lincosamides show poor activity due to both exclusion and efflux mechanisms [25]. More importantly, this intrinsic resilience forms the scaffold upon which acquired resistance genes, can act synergistically, amplifying the threat these organisms pose in nosocomial environments. Of additional concern is the growing emergence of carbapenemase-producing ECC [26].

## 3. Carbapenemases: Classification and Genetic Context

**Figure 1:**
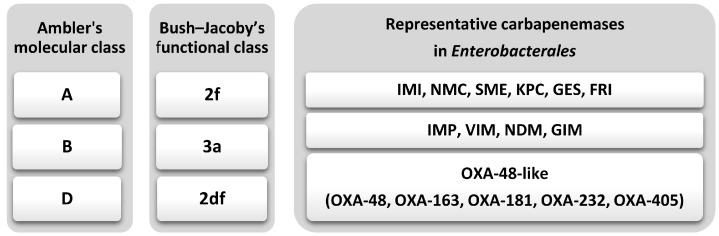
Classification of carbapenemases.

The primary mechanism conferring carbapenem resistance in *Enterobacterales* involves the enzymatic degradation of carbapenems by carbapenemase enzymes, which effectively neutralize these critical β-lactam agents. Carbapenemases exhibit a broad substrate spectrum, extending beyond carbapenems to include other β-lactam antibiotics, and display substantial mechanistic diversity [27]. Over four decades ago, Ambler described structural characteristics of β-lactamases, classifying carbapenemases into three distinct molecular classes, A, B and D, based on amino acid sequence and the nature of their active site (Figure 1) [28]. Beside this molecular classification, a Bush–Jacoby’s categorization focused on functional properties of β-lactamases based on their hydrolytic activity and resistance to various β-lactamase inhibitors [27]. According to this functional classification, carbapenemases belong to three groups, 2f, 2df, and 3a (Figure 1) [29].

Ambler classes A, B, and D carbapenemases are primarily differentiated by the structural and chemical properties of their active sites, the underlying mechanisms of β-lactam hydrolysis, and their distinct profiles of inhibitor susceptibility.

Class A (serine carbapenemases) use a serine residue at the active site for hydrolysis of the β-lactam ring. They show broad-spectrum hydrolytic activity, including all β-lactams and have been variably inhibited with clavulanic acid or tazobactam [29]. A newer generation of inhibitors, such as avibactam, relebactam, durlobactam and zidebactam, are highly effective against class A carbapenemases [30]. Chromosomally encoded serine carbapenemases IMI-1 (Imipenem-Hydrolyzing β-lactamase-1), NMC-A (Non-Metallo Carbapenemase-A) and SME (*Serratia marcescens* enzyme) were the first carbapenemases described in *Enterobacterales* [27]. IMI-1 and NMC-A were detected in *E. cloacae* clinical isolates in the early 1980s in the USA and in 1990 in France, respectively, while SME was found in *S. marcescens* isolates in 1982 in the United Kingdom [27,31]. These three types of class A carbapenemases exhibit considerable structural similarity. Notably, IMI and NMC-A share more than 97% amino acid sequence identity and are grouped under the IMI-like enzyme family, for which approximately 20 variants have been identified to date [32]. In contrast, SME enzymes are more divergent, demonstrating approximately 70% similarity to the former two, but with no major functional differences [27,31]. The structural resemblance results into a comparable hydrolytic spectrum, encompassing activity against penicillins, early-generation cephalosporins, aztreonam, and carbapenems, while displaying limited efficacy against cephamycins and extended-spectrum cephalosporins such as ceftazidime [27,33]. These enzymes are often co-expressed with other chromosomal β-lactamases, including AmpC-type cephalosporinases, and their expression can be induced by agents such as imipenem and cefoxitin [31]. Due to chromosomal localization of the IMI-like enzymes, there was limited spreading of these carbapenemases [27].

In contrast, plasmid-encoded serine carbapenemase KPC (*Klebsiella pneumoniae* carbapenemase), first reported in the United States in 1996, is recognized as the most widespread type among *Enterobacterales* [34]. KPC belongs to the so-called “Big Five” carbapenemases, which are distinguished by their major clinical relevance and broad epidemiological spread [35]. Shortly after the initial identification of KPC-1, additional variants such as KPC-2 and KPC-3 were reported. To date, approximately 150 KPC variants have been described, all originating from mutations of *bla*_KPC-2_ or *bla*_KPC-3_ genes [36]. KPC-producing strains exhibit hydrolytic activity against all β-lactam antibiotics and are frequently multidrug resistant. The main reason is that *bla*_KPC_ gene is primarily carried by the highly conserved transposon Tn*4401*, typically co-located on the same plasmid with other resistance genes, including those for extended-spectrum β-lactamases (ESBLs), aminoglycosides, and fluoroquinolones [32,35,37]. The most prominent plasmid types associated with Tn*4401* are ColE1, IncA/C, IncF, IncI2, IncR and IncX [38]. Another plasmid-mediated, serine carbapenemase is GES (Guiana extended spectrum), initially identified in 1998 in a *K. pneumoniae* isolate in France [27,33]. Originally classified as an ESBL, due to its ability to hydrolyze penicillins and broad-spectrum cephalosporins, the classification of GES enzymes was later revised following the emergence of variants capable of hydrolyzing imipenem, thus reclassifying them as carbapenemases [27,33]. Among more than 40 GES variants, those with G170N substitution are capable of carbapenem hydrolysis [33]. One of the latest recognized class A carbapenemase is FRI (French resistance to imipenem), detected in an *E. cloacae* isolate in Paris, France in 2015 [33]. Alongside IMI, NMC-A and SME, FRI carbapenemase comprise in a group of “minor carbapenemases”, showing a similar hydrolytic spectrum [33]. The *bla*_FRI-1_ gene has been linked to a LysR-family transcriptional regulator and is typically located on plasmids of the IncFII/IncR type [33]. The plasmid-borne nature of FRI enzymes and growing number of variants (12 described to date) contributes to their increased clinical relevance, positioning them as emerging yet frequently under-recognized carbapenemases of growing public health concern [38].

Ambler’s class B metallo-β-lactamases (MBLs) are zinc-dependent enzymes capable of hydrolyzing all β-lactams except monobactams [29]. The major MBL types are IMP (imipenem-resistant), VIM (Verona integron-encoded metallo-β-lactamase) and NDM (New Delhi metallo-β-lactamase), belonging to the so-called “Big Five” carbapenemases. Their structural properties confer resistance to clinically used β-lactamase inhibitors (clavulanic acid, tazobactam, avibactam), though they can be inhibited in vitro by metal chelators such as ethylenediaminetetraacetic acid (EDTA) [39]. Despite the fact that avibactam alone does not show activity against MBLs, an aztreonam–avibactam combination represent promising therapy solution for MBL-producing *Enterobacterales* [30]. Newer inhibitors zidebactam, nacubactam, or components in clinical development such as taniborbactam and QPX7728, are effective against MBLs [30]. Although chromosomally MBLs were among the earliest β-lactamases to be characterized, their clinical significance remained relatively limited for some time [32]. The discovery of a novel plasmid-mediated MBL in Japan during the early 1990s raised concerns about the potential for rapid global dissemination of transferable MBLs. IMP was the first plasmid-mediated carbapenemase identified in *Enterobacterales*, detected in *S. marcescens* in Japan in 1991 [40]. Since then, about 88 IMP variants have been assigned as IMP type carbapenemase [38]. The *bla*_IMP_ gene is primarily associated with class 1 and class 3 integrons, which are integrated into a variety of plasmid types, most notably those belonging to the IncL/M and IncA/C incompatibility groups [41]. IMP carbapenemase is less widespread than other MBL types, being endemic in East Asia [32,35]. VIM was initially discovered in *P. aeruginosa* in 1996 and was less commonly linked to carbapenem resistance in *Enterobacterales* compared to other MBLs [32]. However, by the early 2000s, VIM-producing *K. pneumoniae* had become endemic in Southern Europe and South Asia [5,32,35]. There are more than 40 variants of VIM enzyme, with the most clinically relevant clusters being the VIM-1-like, VIM-2-like and VIM-7-like groups [38]. The *bla*_VIM_ gene is predominantly located within class 1 integrons, which are frequently linked to transposons and may be integrated either into the bacterial chromosome or plasmid backbones (e.g., IncA/C or IncN), facilitating broad dissemination [38]. Unlike IMP and VIM, NDM is the most prevalent and widely distributed MBL in *Enterobacterales*. First identified in Sweden in 2008 in a *K. pneumoniae* isolate from a patient with links to India [42], NDM is now endemic in India, the Balkan region, Northern Africa, and the Arabian Peninsula [32,35,38]. Studies have shown extensive genetic diversity in the *bla*_NDM_ background, with no association to a specific clone, species, or plasmid type, facilitating its global spread [32,35]. To date, 24 distinct NDM variants have been identified, across more than 60 bacterial species. More than 350 plasmids are associated with *bla*_NDM_ gene, with approximately 20 distinct plasmid types identified in *Enterobacterales*, among which IncX3 is the most frequently encountered [38]. GIM (Germany Imipenemase) type of MBL was first describe in 2009 in *S. marcescens* isolates from Germany, where it has remained endemic [33]. It is classified among the so-called “minor carbapenemases”, with only two variants identified thus far. GIM is part of a group of less commonly encountered MBLs, which includes KHM (Kyorin Hospital Metallo-β-lactamase), TMB (Tripoli Metallo-β-lactamase), SFH (*Serratia fonticola* carbapenem hydrolase), AIM (Adelaide Imipenemase), and LMB (Linz Metallo-β-lactamase) [33]. The *bla*_GIM−1_ is part of class 1 integron and carried by various conjugative plasmids, posing a significant risk of dissemination beyond its current niches in Germany [33].

OXA (oxacillinase) enzymes, classified as Ambler class D β-lactamases, were initially identified based on their ability to hydrolyze oxacillin, methicillin, and cloxacillin. These enzymes exhibit minimal activity against extended-spectrum cephalosporins and carbapenems and are not inhibited by EDTA or clavulanic acid [43]. Certain novel β-lactamase inhibitors have demonstrated the ability to inhibit carbapenem hydrolysis mediated by OXA-type carbapenemases. Among them, avibactam is already in clinical use, while others, such as nacubactam, CTB/VNRX-5236, QPX7728, and LN-1-255, are currently under development [30]. Although they represent the most diverse group of carbapenemases, with more than 750 OXA variants, only a small subgroup exhibits hydrolytic activity against carbapenems, typically observed in conjunction with additional resistance mechanisms, leading to a carbapenem-resistant phenotype [32,43]. The most significant OXA-type carbapenemase in *Enterobacterales* is OXA-48, first identified in a *K. pneumoniae* isolate from Turkey in 2003 [35]. Since then, it has rapidly spread beyond its endemic regions in Turkey, the Middle East (Lebanon, Jordan, Oman, Iran, and Saudi Arabia), and North Africa (Morocco, Algeria, Tunisia, and Egypt) [35,37,43]. In addition to OXA-48, several closely related variants form the OXA-48-like group, including OXA-162, OXA-181, OXA-204, OXA-232, OXA-244, and OXA-405 [38]. Unlike NDM, the global dissemination of OXA-48-positive clones is primarily driven by a specific genetic element, Tn*1999* on the IncL/M self-conjugative *pOXA-48a*-like plasmid [35,43].

## 4. Epidemiology of Carbapenemase-Producing *Enterobacter* spp. in Europe

**Figure 2:**
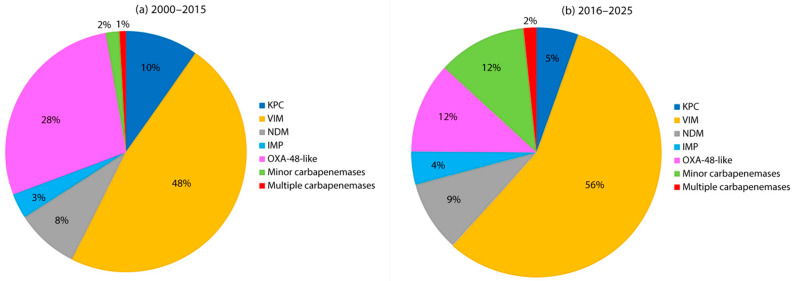
Carbapenemase types among *Enterobacter* spp. isolates in Europe. (**a**) 2000–2015, (**b**) 2016–2025.

**Figure 3:**
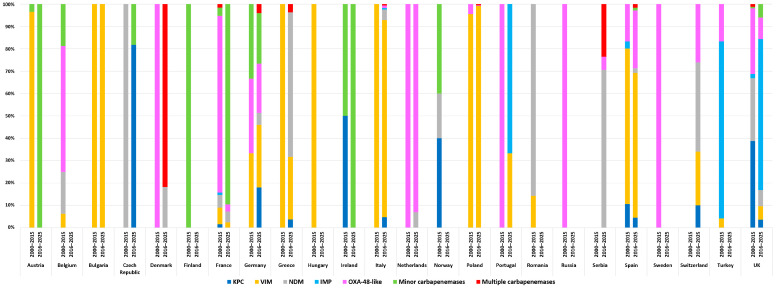
Temporal and geographical distribution of carbapenemases in *Enterobacter* spp. (2000−2015 vs. 2016−2025).

Carbapenemase-producing *Enterobacterales* (CPE) have emerged as a major public health concern worldwide, and *Enterobacter* spp., particularly members of the ECC, represent a significant but often underrecognized reservoir in Europe. Since the early 2000s, plasmid-mediated carbapenemases have been increasingly detected among *Enterobacter* isolates in both outbreak and sporadic settings, mirroring the broader epidemiological trends seen in *K. pneumoniae* and *E. coli*. The most prevalent enzymes identified in European *Enterobacter* spp. include, VIM, OXA-48-like, KPC, and NDM carbapenemases, though their distribution is strongly influenced by geography and clonal background (Figure 2 and Figure 3, Supplementary Table S1).

When analyzing temporal trends, two distinct epidemiological phases can be observed, before and after 2015, each characterized by a different distribution of dominant carbapenemase types (Figure 2 and Figure 3). In the first surveillance period (2000–2015), VIM-type enzymes were already dominant, accounting for nearly half of carbapenemase-producing *Enterobacter* spp., with OXA-48-like carbapenemases representing the second most frequent group (28%). KPC and NDM were present at lower levels (10% and 8%, respectively), but still contributed to the early diversity of resistance mechanisms. After 2015, the distribution shifted further: VIM consolidated its dominance, rising above 55%, while OXA-48 and KPC both declined substantially, to around 12% and 5%, respectively. At the same time, NDM slightly increased its prevalence and minor carbapenemases emerged as an additional contributor, reflecting an evolving epidemiological pattern. These changes underline the fluid dynamics of carbapenemase dissemination, with certain determinants gaining regional advantage while others decline.

**Figure 4:**
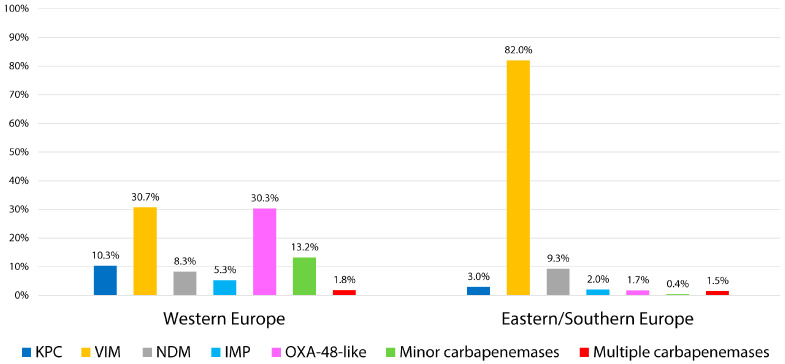
Distribution of carbapenemase types among *Enterobacter* spp. in Western vs. Eastern/Southern Europe.

Clear regional differences were observed in the distribution of carbapenemase types among *Enterobacter* spp. isolates across Europe. In Eastern and Southern Europe, isolates were predominantly associated with VIM-type carbapenemases, with NDM representing an additional but regionally relevant enzyme. In contrast, Western Europe showed a more heterogeneous profile, where VIM and OXA-48-like enzymes were the most frequent, followed by KPC, NDM, and IMP producers (Figure 4). These findings highlight distinct regional epidemiological trends in the dissemination of carbapenemase genes, shaped by local antimicrobial use, infection control policies, and healthcare connectivity across Europe.

### 4.1. IMI-like Enzymes

IMI-like carbapenemases, encompassing IMI and NMC-A variants, are among the earliest class A serine carbapenemases identified in *Enterobacter* spp. but remain rare and frequently underrecognized due to their chromosomal localization and atypical resistance phenotype. The first European case of an NMC-A-producing *E. cloacae*, later reclassified as *E. asburiae*, was reported in Paris, France in 1990 [44]. The isolate carried the *bla*_NMC-A_ gene integrated into the chromosome within a novel 29-kb element, EludIMEX-1, mobilized through XerC/XerD site-specific recombination, representing a previously undescribed mechanism of carbapenemase gene integration [45]. Subsequent sporadic detections were recorded across Europe. Between 2008 and 2011, the Finnish National Institute for Health and Welfare identified three *E. cloacae* isolates carrying IMI-1, IMI-2, and NMC-A-type carbapenemases, respectively, mostly in patients with a history of hospitalization abroad, suggesting importation rather than local acquisition [46]. In Ireland, the first IMI-1-producing *E. asburiae* was detected in 2013 [47]; the isolate exhibited an unusual phenotype with resistance to carbapenems, colistin, and fosfomycin, while phenotypic carbapenemase tests remained negative, underscoring diagnostic challenges. Later, IMI-2-producing *E. cloacae* were detected in healthy carriers, highlighting silent intestinal colonization and the possible influence of environmental exposure, such as recreational water use [48].

France has provided the most comprehensive insight into IMI-type enzymes in Europe. The first French clinical isolate, recovered in 2011 from a case of ventilator-associated pneumonia, carried chromosomal *bla*_IMI-1_ regulated by the LysR-type activator *imiR*, conferring high-level resistance to imipenem while maintaining susceptibility to extended-spectrum cephalosporins [49]. Surveillance by the French National Reference Center (2014–2022) documented 112 non-duplicate IMI-producing ECC isolates, obtained from clinical and screening specimens across multiple hospitals [50]. Chromosomal variants (IMI-1, IMI-4, IMI-12, IMI-13) predominated in *E. cloacae* subsp. *cloacae*, while plasmid-encoded variants (IMI-2, IMI-6, IMI-17, IMI-19, IMI-25–27) were associated with *E. asburiae* and *E. ludwigii* and carried on IncFII(Yp) plasmids, suggesting interspecies transfer through conjugative elements [50]. A remarkable regional outbreak occurred between 2015 and 2017 in Mayotte and La Réunion, where 18 clonally related *E. cloacae* ST820 isolates carried chromosomal *bla*_IMI-1_ within the EcloIMEX-8 element inserted at the *tRNA^Phe^* locus, accompanied by *imiR* and exhibiting high-level imipenem resistance but residual susceptibility to extended-spectrum cephalosporins [51].

In Spain, both sporadic and clustered cases have been documented. In 2021, *E. ludwigii* carrying plasmid-borne *bla*_IMI-6_ on an IncFIIY conjugative plasmid was recovered from a rectal swab of an elderly patient under lymphoma treatment [52], while in 2022 a nosocomial cluster of IMI-1-producing *E. ludwigii* ST1677 was identified in Gran Canaria, with all isolates clonally related and harboring chromosomal *bla*_IMI-1_ [53]. In Norway, between 2007 and 2014, a single case of IMI-9-producing ECC was reported; the isolate, linked to travel abroad, tested negative in the Carba NP assay, again emphasizing the limitations of routine phenotypic methods [54]. The Czech Republic reported in 2016 an *E. asburiae* strain carrying plasmid-mediated *bla*_IMI-2_, detected during routine screening and demonstrating resistance to imipenem, aztreonam, and colistin but susceptibility to cefepime and piperacillin–tazobactam [55]. Soon after, Austria described a human clinical isolate of *E. mori* carrying *bla*_IMI-2_ on an IncFII plasmid and belonging to a novel sequence type, suggesting environmental or zoonotic crossover [56]. In Scotland, an IMI-producing *E. cloacae* isolate, recovered in 2019 from the bloodstream of a patient undergoing chemotherapy for metastatic breast cancer, further underscored the clinical significance of these enzymes in vulnerable populations [57].

Altogether, European experience with IMI-like enzymes reveals two primary dissemination routes: chromosomal integration linked to healthcare-associated infections and localized clonal persistence (as observed in France, Spain, and Norway), and plasmid-mediated acquisition facilitating horizontal gene transfer and occasional emergence in the community or environmental reservoirs (as seen in Ireland, the Czech Republic, and Austria). Their low phenotypic expression, variable susceptibility profiles, and frequent failure of standard screening assays make IMI-type carbapenemases difficult to detect, underscoring the importance of molecular diagnostics and genomic surveillance for accurate identification and effective infection control.

### 4.2. KPC

KPC-type carbapenemases, though less prevalent in *Enterobacter* spp. than in *K. pneumoniae*, have been repeatedly reported across Europe, typically linked to imported cases and plasmid-mediated dissemination. The first documented European case of a KPC-producing *E. cloacae* occurred in France in 2002, involving a patient previously hospitalized in Israel [58]. Both *E. coli* and *E. cloacae* isolates carried *bla*_KPC-2_ on transferable plasmids, highlighting early interspecies plasmid transfer. By 2011, sporadic and small cluster cases involving *E. cloacae* and *E. aerogenes* (now *K. aerogenes*) were documented, with *bla*_KPC-2_ and *bla*_KPC-3_ embedded in Tn*4401* on IncFII or IncN plasmids [59,60]. These plasmids also frequently harbored additional resistance determinants (*bla*_SHV-12_, aminoglycoside-modifying enzymes), contributing to extensively drug-resistant phenotypes. Surveillance between 2012 and 2014 confirmed low but persistent detection of KPC among French *Enterobacterales* (1.8% of CPE), with occasional autochthonous cases and most isolates linked to prior hospitalization abroad [60].

In Spain, *Enterobacter* spp. harboring KPC were first recorded in 2012, initially rare but associated with multidrug resistance. Early isolates, including *E. cloacae* and *E. asburiae*, carried *bla*_KPC-2_ or *bla*_KPC-3_, frequently coexisting with *bla*_VIM-1_ or *bla*_OXA-48_, located on multireplicon plasmids (IncFII, IncN, IncR) [61,62,63]. These isolates belonged to diverse STs (ST66, ST78, ST101, ST114) and exhibited extensive resistance, including to tigecycline and colistin [62]. Later studies (2013–2018) confirmed polyclonal distribution of *bla*_KPC_, mainly *bla*_KPC-2_, with persistent detection in *E. hormaechei*, *E. roggenkampii*, and *E. kobei*, often in hospital sink environments, reflecting silent environmental persistence [64,65].

The United Kingdom reported its first *E. cloacae* carrying *bla*_KPC-4_ in 2003, associated with a patient returning from the United States [57]. By 2007, sporadic detections of *bla*_KPC-2_ and *bla*_KPC-3_ followed, leading to national genomic surveillance (2009–2014) which identified 26 ECC isolates among 604 *bla*_KPC_-positive samples [66]. These genes, mostly within Tn*4401a*, were carried on IncFII, IncR, or IncN plasmids, often co-harboring *bla*_CTX-M-15_ and aminoglycoside resistance genes. The study confirmed polyclonal spread and plasmid-driven transfer, including participation of *Enterobacter* spp. in the large Manchester KPC outbreak. Further reports (2014–2017) documented *E. cloacae* producing KPC-2 or KPC-3 in Scottish hospitals, mainly linked to prior international healthcare exposure, though local hospital transmission was also confirmed [67].

In Central and Eastern Europe, *Enterobacter* spp. carrying KPC have been reported in several countries. In the Czech Republic, the first isolates appeared in 2009, mostly among repatriated patients from Greece and Italy, and later expanded to 18 ECC isolates detected nationwide in 2018–2019 [68]. These strains carried *bla*_KPC-2_ on Tn*4401a* located on IncR or IncN plasmids, sometimes co-harboring *bla*_VIM-4_ or *bla*_CTX-M-15_. Germany’s surveillance (2013–2019) revealed that most KPC-2-positive isolates, including *Enterobacter* spp., carried a unique IncN [pMLST15] plasmid with a non-Tn*4401* element (NTEKPC-Y), stable across multiple species and sequence types [69]. In Italy, a known KPC-endemic region, *E. cloacae* isolates carrying *bla*_KPC-2_ have been increasingly recovered from clinical and colonization samples since 2018, especially in ICUs of southern hospitals [70]. In Ireland, *E. cloacae* harboring *bla*_KPC-3_ on IncN plasmids were first reported in 2014–2015, mostly linked to patients previously hospitalized abroad, without evidence of local transmission [71]. Poland reported polyclonal endemic spread of *bla*_KPC-2_ among ECC isolates, with *E. cloacae* and *E. hormaechei* belonging to diverse STs (ST66, ST89, ST90, ST114), carrying *bla*_KPC-2_ on Tn*4401a*-bearing IncN or IncFIIK plasmids [72]. Occasional imported cases were also detected in Norway, with no secondary spread [73].

Altogether, KPC-producing *Enterobacter* spp. in Europe illustrate a mosaic of sporadic introductions, localized outbreaks, and silent plasmid persistence. Although still less frequent than VIM or OXA-48 producers, *bla*_KPC_-positive *Enterobacter* spp. represent a growing concern due to their association with high-risk clones (*E. cloacae* ST78, *E. hormaechei* ST66, ST171) and epidemic plasmids (IncFII, IncN, IncR). Their persistence across healthcare networks underscores the need for enhanced genomic surveillance, strict infection control, and coordinated One Health monitoring of potential environmental reservoirs.

### 4.3. GES

The first documented GES-producing ECC isolate in Europe emerged in the Czech Republic in 2016 [21]. A carbapenem-resistant *E. cloacae* ST252 (isolate Ecl-35771cz) was recovered from a patient’s leg wounds. Phenotypic tests failed to detect carbapenemase activity, but a mass-spectrometry hydrolysis assay and PCR/sequencing identified the *bla*_GES-5_ gene. WGS showed that *bla*_GES-5_ was the first cassette of a novel class-1 integron, In1406, located on a 6.9-kb ColE2-like plasmid pEcl-35771cz. The integron also carried a novel *aadA15* allele and was inserted into a mobilizable plasmid backbone. The patient had no travel history abroad and subsequent surveillance detected no additional GES-positive isolates, suggesting this was a sporadic event. Further, evidence for environmental persistence and plasmid evolution came from a Czech hospital survey in 2020 [74]. Two ECC isolates (ST837 and a novel ST1622) from sink surfaces carried pEcl-35771cz-like plasmids containing two *bla*_GES_ cassettes. In one plasmid (In2079), the cassettes encoded *bla*_GES-1_ and *bla*_GES-5_; in the other (In2081), there were two copies of *bla*_GES-5_ [74].

In a London hospital, an unexpected *E. cloacae* ST66 isolate carrying GES-5 was found in a patient [75]. Routine tests missed the carbapenemase, but sequencing showed it harbored the same 8.3-kb IncQ plasmid that had caused an earlier *K. oxytoca* cluster and was later detected in *E. coli* and hospital wastewater [75]. This highlights that GES-5 plasmids can silently jump between species and that molecular testing is essential to identify GES-positive *Enterobacter* spp. in time.

The dataset of Kazmierczak et al. reported that among meropenem-non-susceptible *Enterobacterales* collected in Europe, there was one isolate of *Enterobacter* spp. carried GES-6 together with CTX-M-15 and a TEM-type β-lactamase. No outbreak or further spread of GES-6 was documented, but the finding highlights that *Enterobacter* spp. can occasionally harbor uncommon GES variants [76].

### 4.4. FRI

The first documented case of an FRI-1-producing *E. cloacae* in Europe was reported in France in 2015 [77]. The isolate, referred to as DUB, was recovered from a urine sample of a hospitalized patient with prior travel to Switzerland. It exhibited resistance to carbapenems, penicillins, and aztreonam, while remaining susceptible to expanded-spectrum cephalosporins. Molecular analysis identified a novel Ambler class A β-lactamase, named FRI-1, encoded by the *bla*_FRI-1_ gene located on a 110-kb non-self-conjugative plasmid. The gene was under the control of a LysR-type transcriptional regulator (FriR), conferring inducible resistance.

In the United Kingdom, an ECC isolate, designated NRZ-587, was the first to be identified as harboring the FRI-2 variant [78]. The isolate was obtained in 2016 from a rectal screening swab of a patient with no travel history, suggesting local acquisition. The isolate exhibited resistance to carbapenems but retained susceptibility to cephalosporins and aminoglycosides. WGS confirmed *bla*_FRI-2_, located on a 108-kb IncF/IncR plasmid. The gene showed 97% nucleotide identity with *bla*_FRI-1_, and lacked upstream IS elements present in FRI-1, indicating a distinct genetic environment. The isolate belonged to a novel sequence type, ST829.

In Germany, an FRI-3-producing ECC isolate, designated NRZ-28021, was recovered in 2016 from a rectal swab of a patient in an ICU in Southern Germany [79]. Sequence analysis identified *bla*_FRI-3_, sharing 91–96% amino acid identity with other FRI variants. The gene was located on a 111-kb IncFII plasmid containing conjugative transfer elements, although conjugation was unsuccessful. As with FRI-1, the gene was adjacent to a LysR-type regulator. Kinetic studies showed that FRI-3 had the same substrate spectrum as FRI-1, though with generally lower catalytic efficiency.

### 4.5. IMP

Reports of IMP-type MBLs in *Enterobacter* spp. across Europe remain sporadic but significant, revealing both clinical and environmental reservoirs. The earliest cases were described in Turkey during the early 2000s within the SENTRY Antimicrobial Surveillance Program (2000–2004), where 19 *E. cloacae* isolates carrying *bla*_IMP-1_ were identified from blood, urine, and wound infections in Ankara and Istanbul. PFGE showed three distinct clones, indicating localized but genetically diverse dissemination, with reduced susceptibility to imipenem and meropenem linked to combined carbapenemase production and porin loss [80]. The first domestically acquired IMP-producing *E. cloacae* in the United Kingdom was reported from Addenbrooke’s Hospital, Cambridge, based on isolates recovered between 2008 and 2009. All three isolates, two from blood and one from urine, carried an IMP-1-like gene confirmed by sequencing, showed resistance to meropenem and other β-lactams, and were clonally identical though epidemiologically unrelated [81]. In Spain, a nationwide study conducted in 2013 found five *Enterobacter* isolates among 379 carbapenemase producers, harboring *bla*_IMP-13_ or *bla*_IMP-22_ and exhibiting resistance to carbapenems, aztreonam, and third-generation cephalosporins but preserved susceptibility to colistin and amikacin [82].

France reported occasional detections of IMP-positive *Enterobacter* isolates, primarily from its overseas territories such as New Caledonia, during surveillance between 2012 and 2014. Although molecular typing was limited, *bla*_IMP_ was confirmed by PCR, and isolates displayed reduced susceptibility to several β-lactams but retained activity against fosfomycin and colistin [60]. In Central and Eastern Europe, Poland’s national surveillance from 2006 to 2019 identified 934 non-duplicate carbapenemase-producing *Enterobacterales* with VIM or IMP genes, including 375 *Enterobacter* isolates (mainly *E. hormaechei*). Four (1.1%) carried *bla*_IMP-19_, located within the novel integron In2241 and inserted into plasmids (IncHI2, IncA, IncF) or chromosomal islands. These isolates originated mostly from urine, wound, and bloodstream infections, showed high-level carbapenem resistance, but remained susceptible to colistin and amikacin [83].

The most recent and molecularly detailed report originated from Italy, where *E. cloacae* ST837 carrying both *bla*_IMP-19_ and *mcr-4.3* was identified in 2023 during routine screening [84]. The isolate was resistant to cefiderocol but susceptible to colistin, with genomic analysis revealing seven plasmids, one (p4-FZ47, 60 kb) harboring *bla*_IMP-19_ within an integron-like structure flanked by IS26 elements. The patient had no travel history or prior antibiotic use, suggesting local acquisition. In Portugal, a 2023 outbreak investigation detected *E. hormaechei* ST133 producing IMP-22 from environmental sources such as sinks and cleaning trolleys, indicating clonal spread and environmental persistence within hospital infrastructure [85].

Across Europe, *bla*_IMP_ genes are almost universally located within class 1 integrons, commonly embedded in Tn*21*-like transposons or carried on broad-host-range plasmids (IncL/M, IncC, IncN) and less frequently on narrow-range plasmids (IncFII(K)). Comparative genomic analysis further demonstrated both interspecies mobility and regional confinement of these elements, with IMP-encoding IncFII(K) plasmids largely restricted to Asia, while IncL/M and IncC plasmids have disseminated globally [86]. Collectively, European evidence suggests that IMP-type carbapenemases in *Enterobacter* spp. remain rare but increasingly diverse, often emerging through environmental persistence and plasmid exchange, underscoring their potential for silent spread within healthcare ecosystems.

### 4.6. VIM

VIM-type MBLs represent one of the most widespread carbapenemase families among *Enterobacter* spp. in Europe, with origins tracing back to southeastern Europe. The earliest documented case was reported in Turkey, where *E. cloacae* carrying *bla*_VIM-5_ was recovered before 2002 [87]. The gene was located in a class 1 integron on a ~23-kb plasmid and encoded a metalloenzyme inhibited by EDTA, marking both the first report of VIM-5 and the first detection of a VIM enzyme in *E. cloacae*. This case highlighted Turkey as an early reservoir for MBLs within the ECC.

In Greece, *bla*_VIM-1_-producing *E. cloacae* was first detected in 2002, initially as isolated cases and later in ICU outbreaks [88,89]. These isolates carried *bla*_VIM-1_ within class 1 integrons on conjugative plasmids, showed multidrug resistance with preserved susceptibility to colistin and tigecycline, and demonstrated clear evidence of clonal hospital dissemination. By the early 2010s, VIM-1 had become endemic in Greek hospitals, with *Enterobacter* spp. frequently isolated from colonized or infected ICU patients [90]. The most recent multicenter surveillance (2020–2023) confirmed that *bla*_VIM-1_ remains the predominant carbapenemase gene among *Enterobacter* spp. in Greece, particularly in bloodstream infections [91].

Spain was among the first western European countries to report VIM-producing *Enterobacter*, with hospital outbreaks documented in Madrid (2005–2006) and Santiago de Compostela (2009) involving *E. cloacae* carrying *bla*_VIM-1_ on IncHI2 or IncI1 plasmids [92,93]. Co-occurrence with *bla*_CTX-M-9_ and other ESBLs was frequent, reflecting extensive plasmid-mediated exchange [94]. A nationwide study spanning 2005–2018 confirmed *bla*_VIM-1_ as the most prevalent carbapenemase in *Enterobacter* spp., particularly in *E. roggenkampii*, *E. ludwigii*, and *E. cloacae* [65]. More recent genomic data (2024) from southern Spain identified multiple clones (*E. cloacae* ST78, ST111, ST90, ST311, and ST22) carrying *bla*_VIM-1_ or *bla*_VIM-63_, demonstrating ongoing clonal diversification [95].

Italy followed soon after, reporting *bla*_VIM-1_-positive *E. cloacae* in 2008 [96]. Subsequent studies confirmed hospital persistence of VIM-1 within integron-bearing IncN and IncHI2 plasmids and frequent involvement of sequence types ST78 and ST114 [97,98,99,100]. A national survey in 2024 reaffirmed *bla*_VIM-1_ as the dominant carbapenemase among *Enterobacter* spp., with emerging evidence of chromosomal integration favoring stable maintenance [101].

In France, *bla*_VIM-1_ and *bla*_VIM-2_ were identified in ECC isolates during national surveillance in the late 2000s [59,102]. These genes, typically carried on IncHI2 plasmids within integrons (In110, In416), were often linked to prior hospitalization in Mediterranean countries but also detected in domestic cases, indicating secondary local transmission.

Central and Eastern Europe have also played a major role in the spread of VIM-type enzymes. In Poland, *Enterobacter* spp. (predominantly *E. hormaechei*) accounted for more than half of 934 VIM/IMP-producing *Enterobacterales* isolates collected from 246 hospitals between 2006 and 2019 [83]. *bla*_VIM-1_, *bla*_VIM-2_, and *bla*_VIM-4_ were embedded in class 1 integrons (In238, In916, In1008) located on broad-host-range plasmids (IncHI2, IncA/C, IncFII). Clonal analysis identified dominant sequence types ST90 and ST89, supporting combined clonal and horizontal dissemination. In Croatia, VIM-1-producing *E. cloacae* were first detected between 2011 and 2012, mainly from blood and urine, and later became established as endemic nosocomial pathogens [103,104]. Austria (2006–2010) reported 28 *E. cloacae* isolates carrying *bla*_VIM-1_, forming three PFGE-defined clusters that reflected both outbreaks and long-term persistence [105], while in Hungary, a neonatal ICU outbreak in 2010 involved *E. cloacae* co-producing VIM-4 and SHV-12 [106].

Collectively, VIM-producing *Enterobacter* spp. are now widespread throughout Europe, with VIM-1 as the predominant variant and *E. cloacae* and *E. hormaechei* as the leading hosts. Their dissemination has been driven by both clonal expansion and interspecies plasmid transfer via class 1 integrons and IncHI2/IncN plasmids. From the first VIM-5 case in Turkey to the endemic VIM-1 persistence in southern and eastern Europe, these findings underscore the entrenched nature of VIM carbapenemases and the ongoing need for molecular surveillance and coordinated infection control across the continent.

### 4.7. NDM

The emergence and dissemination of NDM-type carbapenemases in the ECC across Europe over the past 15 years have become an increasing clinical and epidemiological concern. The earliest European detection occurred in the United Kingdom in 2008, where an *E. cloacae* isolate carrying *bla*_NDM-1_ was identified in a patient recently hospitalized in the Indian subcontinent [67,107,108]. The gene was plasmid-borne on IncF or IncA/C plasmids, often co-located with other resistance determinants such as *rmtC*, *aac(6′)-Ib-cr*, *qnrB1*, *qnrS1*, *sul1*, and *sul2*. Subsequent national surveillance (2008–2013) confirmed increasing reports of NDM-positive *Enterobacter* spp., largely imported from South Asia. Later prevalence studies (2017–2018) established NDM as the dominant carbapenemase among *Enterobacterales* in London hospitals, with *E. cloacae* among the most affected species [109].

In continental Europe, the first NDM-producing *E. cloacae* was reported in Belgium in 2010, isolated from a patient with prior hospitalization in Serbia and Kosovo [110]. The strain co-harbored *bla*_CTX-M-15_, *bla*_SHV-12_, *bla*_TEM-1_, *armA*, and *qnrB2-like* genes on IncL/M and IncA/C plasmids, which were transferable to *E. coli*. National data from 2007–2011 documented only sporadic cases, all travel-related [111]. In France, NDM-positive ECC isolates were first recorded between 2010 and 2012, representing 9.7% of carbapenemase producers submitted to the National Reference Centre [102]. These isolates, carrying *bla*_NDM-1_ or *bla*_NDM-5_, were recovered across multiple hospitals, mostly from nosocomial infections, and remained susceptible to colistin and fosfomycin [112].

In southeastern Europe, Croatia reported a 2013 ICU outbreak in Pula involving clonally related *E. cloacae* ST133 isolates harboring *bla*_NDM-1_ on an IncHI2 plasmid alongside *bla*_CTX-M-15_ and *bla*_SHV-12_ [113]. In neighboring Serbia, a study from Belgrade (2016–2017) revealed community-level dissemination, identifying NDM-positive *E. cloacae* in outpatient settings, often co-harboring *bla*_OXA-48-like_ genes [114]. Six clonal lineages were identified, indicating silent spread of high-risk clones outside hospitals.

In Greece, a large outbreak between 2016 and 2022 involved *E. cloacae* ST182 isolates carrying *bla*_NDM-1_ on IncA/C plasmids flanked by ISAba125 and *ble*_MBL_ [115]. The isolates, resistant to all β-lactams, aminoglycosides, and fluoroquinolones but susceptible to colistin and tigecycline, were recovered from multiple clinical sites, indicating prolonged hospital circulation. In Italy, two *E. hormaechei* isolates were detected during the large NDM-1 outbreak in Tuscany (2018–2019), showing typical multidrug-resistant phenotypes but retained susceptibility to colistin and tigecycline [116].

In Spain, NDM-producing ECC were first identified in 2016–2017 in Madrid, involving *E. hormaechei* ST78 isolates harboring *bla*_NDM-7_ on a 45-kb IncX3 plasmid within a composite transposon [117]. These isolates were clonal and resistant to all β-lactams, including novel β-lactam/β-lactamase inhibitor combinations, but showed variable susceptibility to trimethoprim–sulfamethoxazole. Switzerland reported NDM-positive *Enterobacter* spp. between 2013 and 2018, with 21 of 53 carbapenemase-producing isolates carrying *bla*_NDM_, most associated with travel exposure [118].

A unique event occurred in Denmark (2022–2023), where *E. hormaechei* ST79 co-producing NDM-5 and OXA-48-like caused a nationwide outbreak linked to contaminated dicloxacillin capsules [119]. Eleven human cases were confirmed across four regions, and an identical strain was isolated from capsule surfaces. WGS confirmed plasmid-borne *bla*_NDM-5_ and *bla*_OXA-48-like_ on separate elements, highlighting the capacity of ECC to serve as vectors for interspecies and even non-clinical transmission.

Overall, NDM-producing *Enterobacter* spp. remain less common than VIM producers in Europe but show growing geographic reach and genetic diversity. Their spread is primarily plasmid-driven, involving IncF, IncA/C, IncX3, and IncHI2 plasmids, often co-carrying other β-lactamase and aminoglycoside resistance genes. While early introductions were associated with medical travel from South Asia, recent evidence of local outbreaks and even community or pharmaceutical sources underscores the expanding ecological and epidemiological footprint of NDM enzymes in the ECC.

### 4.8. GIM

In Germany, the first identification of GIM-1 in *E. cloacae* was reported in 2012, based on two clinical isolates from Cologne and Frankfurt. Both isolates carried *bla*_GIM-1_, were non-clonal, and showed resistance to ertapenem and ceftazidime, with variable susceptibility to imipenem and meropenem. The *bla*_GIM-1_ gene was located on self-transferable plasmids, including one that replicated in *P. aeruginosa*, indicating a broad host range. Genetic analysis revealed distinct class 1 integron structures, suggesting independent acquisitions. This study demonstrated the first confirmed spread of GIM-1 into *Enterobacterales* beyond *Pseudomonas* spp. in Germany [120].

### 4.9. OXA-48 Like

OXA-48-like carbapenemases have become one of the most prevalent resistance mechanisms in *Enterobacter* spp. across Europe, spreading mainly through IncL/M-type plasmids and transposons such as Tn*1999*. The first documented OXA-48-producing *E. cloacae* isolates were reported in Istanbul in 2010, recovered from blood and cerebrospinal fluid samples [121]. Both carried *bla*_OXA-48_ on a ~70 kb plasmid embedded within Tn*1999* and co-harbored ESBL genes (*bla*_SHV-5_ and *bla*_SHV-2a_), providing early evidence of horizontal transfer of carbapenemase genes into *Enterobacter* spp. Shortly after, in France, an *E. cloacae* isolate from a hospital outbreak at Hôpital Bicêtre, Paris (2011), was identified carrying *bla*_OXA-48_ on a 70-kb IncL/M plasmid (Tn*1999.2*) together with *bla*_SHV-12_ [122]. Although the outbreak primarily involved *K. pneumoniae*, this *E. cloacae* isolate represented the first detection of OXA-48 in France and confirmed interspecies plasmid transfer. Subsequent national surveillance by the French National Reference Center found *E. cloacae* among 6.8% of OXA-48 producers in 2012, with prevalence rising to 9.9% by 2014 [60,123]. Regional studies from 2011–2016 confirmed its association with nosocomial transmission and international travel, particularly to North Africa and Asia [124,125].

In Spain, *Enterobacter* spp. rapidly emerged as notable carriers of OXA-48 following nationwide replacement of VIM by OXA-48 after 2012 [61]. Outbreaks between 2013 and 2014 involved multidrug-resistant *E. cloacae* producing OXA-48 and/or CTX-M-15, with two dominant clones (ST74 and ST66) disseminating across hospitals [126]. A large-scale molecular study in Madrid (2005–2018) confirmed that 18.8% of *Enterobacter* spp. carbapenemase producers carried *bla*_OXA-48_, predominantly in *E. hormaechei* and *E. roggenkampii*, and that the enzyme had become endemic in several hospital networks [60]. Further surveillance confirmed *E. cloacae* as a frequent bloodstream pathogen, with OXA-48 being the predominant carbapenemase and ST114 as a recurrent clone [95].

Belgium was among the earliest countries to report OXA-48-producing *E. cloacae*, with findings from 2011–2012 showing it as the second most common OXA-48-producing species after *K. pneumoniae* [111,127]. A multicountry analysis by Potron et al. (2013) found 10 *E. cloacae* among 107 OXA-48-positive isolates from Europe and North Africa, with more than 90% carrying *bla*_OXA-48_ on an epidemic ~62-kb IncL/M plasmid [128]. Later, novel variants such as OXA-427 were reported, but OXA-48 remained dominant in Belgian hospitals [129].

In Germany, early OXA-48 detections were linked to travel from endemic regions such as Turkey and North Africa [130]. Over time, OXA-48 was increasingly reported in both clinical and screening samples. A hospital study in Hamburg (2013–2016) found *bla*_OXA-48_ in 25% of carbapenem-resistant *E. cloacae*, indicating nosocomial spread [131]. Genomic surveillance between 2019 and 2020 identified *Enterobacter* spp. as the most frequent carbapenemase producers in two tertiary hospitals, with OXA-48 detected in 61% of carbapenemase-positive isolates, mainly *E. hormaechei* and *E. roggenkampii* ST66 [132].

The United Kingdom also documented early OXA-48-positive *E. cloacae* isolates on IncL plasmids [133,134]. In Ireland, Brehony et al. (2019) described seven *E. cloacae* isolates carrying OXA-48-like genes, six with *bla*_OXA-48_ and one with the variant *bla*_OXA-181_, all located on IncL plasmids, frequently alongside *bla*_CTX-M_ ESBLs [135]. The emergence of *bla*_OXA-181_ underscored diagnostic challenges, as standard assays may fail to detect such variants.

In Scandinavia, Denmark reported the novel variant OXA-436 (2013–2015) in *E. asburiae* isolates from multiple hospitals [136]. The *bla*_OXA-436_ gene, located on large 314-kb IncHI2/IncHI2A plasmids, coexisted with *bla*_CTX-M-15_, *qnrA1*, and *aac(6′)-Ib-cr* and was transferable to *E. coli*. Patients lacked travel history, suggesting local emergence, while detection of *bla*_OXA-436_ in multiple *Enterobacterales* species from a single patient confirmed in vivo interspecies plasmid transfer.

Several other European countries have reported sporadic or endemic OXA-48 presence in *Enterobacter* spp. Portugal’s first case was recorded in 2014, co-occurring with *E. coli* [85], Poland identified *bla*_OXA-48_ in *E. cloacae* ST78 and ST89 on plasmids or integrated chromosomally [137,138], Sweden’s NICU outbreak in 2015 demonstrated interspecies transfer from *E. cloacae* ST1584 to other Enterobacterales [139], Croatia detected OXA-48-positive *E. cloacae* in both clinical and environmental sources, carrying IS*1999* and IS*1R* flanking elements typical of Tn*1999* [140], Switzerland reported OXA-48-like producers mainly in western regions bordering France [118], and a Dutch tertiary hospital (2011–2021) documented 12 *Enterobacter* spp. among 84 OXA-48-positive isolates, including one case of chromosomal integration of *bla*_OXA-48_, suggesting potential for stable plasmid-independent maintenance [141].

Overall, OXA-48-like carbapenemases in *Enterobacter* spp. have evolved from sporadic findings to widespread endemicity in European healthcare settings. Their spread is primarily plasmid-mediated, facilitated by epidemic IncL/M plasmids and mobile elements such as Tn*1999*. Common epidemiological features include hospital-associated outbreaks, co-carriage of ESBLs, and silent transmission due to low-level carbapenem resistance. These characteristics underscore the urgent need for improved molecular diagnostics, continuous genomic surveillance, and rigorous infection control to curb further dissemination of these multidrug-resistant pathogens.

### 4.10. Multiple Carbapenemases

Co-production of carbapenemases represents a well-recognized phenomenon, particularly in endemic regions and among MDR bacterial strains. This process results from intensive horizontal gene transfer across bacterial species, documented in both Gram-positive and Gram-negative organisms [32]. Multiple carbapenemase production has been reported in all ESKAPEE pathogens, with a steadily rising global prevalence since 2009. The distribution of species and carbapenemase combinations, however, varies considerably across continents [142].

In Europe, the Mediterranean basin is a hotspot for CPE, making the emergence of double-carbapenemase-producing (DCP) *Enterobacterales* predictable. At a Spanish university hospital, Mateos et al. identified *Enterobacter* isolates co-producing VIM-1 together with KPC-2 or KPC-3 [65]. Several years later (2021–2022), the same institution reported two *E. hormaechei* ST78 isolates carrying VIM-1 and NDM-7, each encoded by distinct mobile genetic elements, thereby heightening the risk of dissemination. Notably, this study showed possible transmission of DCP *Enterobacter* between patients, linked to non-clonal related strains [143]. Italy, an endemic region for KPC-type carbapenemase, has also reported DCP *Enterobacter* spp. A multicentric investigation spanning 2018–2020 in Palermo identified isolates co-harboring KPC and VIM [70]. In Greece, two tertiary hospital isolates (2020–2023) carried VIM-1/KPC and VIM-1/OXA-48 combinations [91]. Similarly, French reports from 2012–2014 described ECC strains producing both VIM-1 and OXA-48 [60,144].

Further north, Poland documented multiregional and interregional spread of *E. hormaechei* subsp. *steigerwaltii* ST90 between 2009 and 2019. Initially producing VIM-4, some isolates subsequently acquired IMP-19, resulting in DCP variants [83]. In the UK, analysis of OXA-48-like carbapenemase producers submitted to PHE’s AMRHAI Reference Unit (2007–2014) revealed two ECC isolates co-producing OXA-48 and NDM-1 [133]. A German prospective study (2019–2020) described *E. roggenkampii* ST96 strains harboring both NDM-5 and OXA-48 [132]. Remarkably, even in Northern Europe—where carbapenem resistance rates are typically low—DCP *E. hormaechei* ST79 strains co-producing NDM-5 and OXA-48 were confirmed in nine patients across Denmark and Iceland (October 2022–January 2023). There were no epidemiological links identified between these patients, though all had received dicloxacillin capsules from the same manufacturer [119].

The Balkan Peninsula, known for NDM endemicity, has also reported DCP *Enterobacterales*. In Belgrade, Serbia, NDM-positive *E. cloacae/asburiae* isolates were identified in community settings (2016–2017), with 23.5% co-harboring NDM and OXA-48 [114]. In Croatia, coproduction of VIM-1 with either OXA-48 or NDM was detected in hospital *E. cloacae* isolates (2013–2014) [104]. A striking observation by Bosnjak et al. in Zagreb revealed two *E. hormaechei* ST200 isolates simultaneously producing three carbapenemases—VIM-2, NDM-1, and OXA-48—originating from two separate ICUs [145].

The co-production of carbapenemases in *Enterobacter* spp. has emerged from a sporadic finding into a widespread event with substantial clinical relevance. Even strains harboring identical carbapenemase combinations often display variable resistance profiles to individual carbapenems. This variability is largely attributable to the presence of multiple plasmids carrying carbapenemase genes in conjunction with additional resistance and virulence determinants. Such complexity significantly complicates the therapeutic management of DCP infections, underscoring the need for individualized treatment strategies [142].

## 5. Conclusions and Future Perspectives

Carbapenemase-producing *Enterobacter* spp. are an increasingly recognized but still underappreciated threat within the spectrum of multidrug-resistant *Enterobacterales*. This review highlights their genetic diversity, capacity for horizontal gene transfer, and involvement in both sporadic cases and large-scale outbreaks across Europe. VIM-type enzymes have emerged as the dominant carbapenemase, while OXA-48-like, KPC, and NDM enzymes continue to circulate at varying regional levels, often coexisting within high-risk clones and epidemic plasmids. The capacity of *Enterobacter* spp. to act as both opportunistic pathogens and silent reservoirs underscores their role in sustaining the carbapenemase epidemic.

The persistence and evolution of these organisms emphasize the urgency of improving surveillance systems. Despite being designated by the WHO as “critical-priority” pathogens, *Enterobacter* spp. remain excluded from major AMR surveillance frameworks such as GLASS. This exclusion hampers early detection and limits evidence-based interventions. In alignment with the WHO Global Action Plan on Antimicrobial Resistance and the 2024 WHO GPPL, it is essential that national health authorities and regional networks incorporate *Enterobacter* spp. into systematic AMR surveillance, laboratory reporting, and infection-prevention strategies.

At the policy level, WHO recommends the implementation of strengthened laboratory capacity, data sharing through GLASS, and integration of the One Health approach across human, animal, and environmental sectors. Incorporating these recommendations would improve detection of carbapenemase-producing *Enterobacter* spp., enable more accurate risk assessment, and support targeted stewardship interventions.

Ultimately, mitigating the silent but growing impact of carbapenemase-producing *Enterobacter* spp. requires coordinated action among clinicians, microbiologists, policymakers, and international health organizations. Only by aligning surveillance, research, and public health policies with WHO guidance can we effectively curb the spread of these high-risk pathogens and preserve the remaining efficacy of life-saving antimicrobials.

## Data Availability

No new data were created or analyzed in this study. Data sharing is not applicable to this article.

## Supplementary Materials

The following supporting information can be downloaded at: https://www.mdpi.com/article/10.3390/antibiotics14101045/s1, Table S1: Characteristics of carbapenemase-producing Enterobacter spp. isolates in Europe (2000–2025).
